# Drug use evaluation of cefepime in the first affiliated hospital of Bengbu medical college: a retrospective and prospective analysis

**DOI:** 10.1186/1471-2334-13-160

**Published:** 2013-04-03

**Authors:** Shi Qingping, Ding Feng, Sang Ran, Liu Yan, Yuan HaoYu, Yu Meiling

**Affiliations:** 1 Department of Pharmacy, The First Affiliated Hospital of Bengbu Medical College, Bengbu, 233004, China; 2 College of Pharmacy, Bengbu Medical College, Bengbu, 233004, China; 3 HeGongYe 416 Hospital, Chengdu, 610051, China

**Keywords:** Cefepime, Clinical pharmacy, Cost-effectiveness, Drug use evaluation

## Abstract

**Background:**

Cefepime is a fourth generation cephalosporin antimicrobial. Its extended antimicrobial activity and infrequent tendency to engender resistance make it popular for the treatment of infections. However, proper use of cefepime has not been studied adequately. In this study, we used a retrospective cohort and a prospective cohort to evaluate the usage pattern, adverse effects and cost-effectiveness of cefepime by conducting a drug use evaluation (DUE) program in the First Affiliated Hospital of Bengbu Medical College, Anhui, China.

**Methods:**

The DUE criteria for cefepime were established by applying literature review and expert consultation, an effective method to promote interventions that will improve patient outcomes and the cost-effectiveness of drug therapy. According to the criteria, we performed a cross-sectional retrospective (cycle A) study on 96 hospitalized patients who received cefepime treatment and a prospective (cycle B) study on 111 hospitalized patients with cefepime treatment intervention. After identifying problems with usage and completing a cefepime use evaluation for cycle A, 2 months of educational intervention among professionals were given and a more effective and rational system of cefepime use was set up. During the 2 months, the lectures were arranged and attendance of prescribers was required.

**Results:**

The data from cycle A showed that the biggest problem was irrational prescription of cefepime; bacterial culture and drug sensitivity tests for cefepime were also not carried out. Following 2 months of educational intervention among professionals, the results for cycle B showed that the correct indication rate was 94.59%, compared with 84.38% in cycle A. Use of bacterial culture and sensitivity tests also improved, by 88.29% in cycle B compared with 65.22% in cycle A. Compared with cycle A, the significantly improved items (P < 0.05) in cycle B were blood examination, liver function monitoring, renal function monitoring, dose and duration, dosing frequency and correct medication combinations.

**Conclusions:**

Cefepime can be used appropriately for the right indications and in a cost-effective way for the majority of patients through educational intervention, including the special precautions that must be followed for appropriate dosing frequency and duration. DUE programs will become one model of hospital pharmacy care and part of the plan for continuous improvements to the quality of health care in China.

## Background

Cefepime is a fourth generation cephalosporin antimicrobial with a wide spectrum of antimicrobial activity, high penetration, and stability against most β-lactamases [1]. Researchers have verified that cefepime tends to be more effective than ceftazidime. Because of its extended antimicrobial activity and infrequent tendency to engender resistance, cefepime is popular for the treatment of infections and is widely used to treat severe nosocomial pneumonia, as well as for empirical treatment of febrile neutropenia, uncomplicated and complicated urinary tract infections, uncomplicated skin and skin structure infections, and complicated intra-abdominal infections [2,3]. However, it should be used judiciously because unnecessary, improper, and prolonged use may lead to the emergence of cefepime-insensitive bacteria and risk decline in its efficacy [4].

The concept of the drug use review (DUR) was raised by the Joint Commission on Accreditation of Healthcare Organizations (JCAHO) [5,6]. Subsequently, the drug use evaluation (DUE) was developed on the basis of the DUR. The DUE is an ongoing, systematic process designed to promote the appropriate and effective use of drugs. The purpose is to detect potential problems and improve drug use. The DUE contains qualitative measures and emphasizes the outcomes and cost-effectiveness of drug therapy. According to the DUE criteria for cefepime published by the American Society of Hospital Pharmacists (ASHP), a cefepime regimen should be restricted to the following indications [7]: 1. Treatment of severe infection caused by suspected gram-negative bacteria or mixed aerobic bacteria (including *Pseudomonas aeruginosa*, except for central nervous system infections) in hospitalized patients, but not used for infection caused by suspected anaerobic bacteria, enterococci or pivmecillinam-resistant staphylococcal infection; and 2. Treatment of the following confirmed infections: urinary tract infections, respiratory tract infections, infections of the skin and subcutaneous tissue; infective endocarditis; osteomyelitis; pathogen infection and bacteremia caused by susceptible organisms sensitive to cefepime, but resistant to other more toxic but less expensive drugs.

To study the current status of cefepime use in the First Affiliated Hospital of Bengbu Medical College, data were collected from patients who received cefepime treatment. In the present study, we performed a historical cohort study (cycle A) on 96 hospitalized patients who received cefepime treatment. Following 2 months of educational intervention among professionals, we performed a prospective (cycle B) study on 111 hospitalized patients with cefepime treatment intervention. Finally, an effective and rational system of cefepime use was set up in the First Affiliated Hospital of Bengbu Medical College.

## Methods

### Study setting

The First Affiliated Hospital of Bengbu Medical College is a 1936-bed tertiary care facility in Anhui, China. The hospital includes all major departments and services, including five pediatric wards and four geriatric wards, hematology and oncology pediatrics, 14 surgical departments, laboratory, X-ray, ultrasound scanner and electrocardiogram, pharmacy, etc. The study was approved by the Department of Health of Anhui Province in China, and the DUE criteria for cefepime and the DUE procedure were approved by the Drug and Therapeutics Committee (DTC) of the hospital.

### Study design

The study duration was divided into two periods (cycle A and cycle B). Cycle A was a cross-sectional retrospective study on 96 hospitalized patients selected because they had received cefepime treatment, performed from June 2011 to October 2011 through access to electronic medical records or medical case records to obtain cefepime use information once patients were discharged from the hospital. After identifying problems with use and completing a cefepime use evaluation for cycle A, 2 months of educational intervention among professionals was given and a more effective and rational system of cefepime use was set up. The 2-month educational intervention was carried out in November and December of 2011. During these 2 months, lectures were arranged and attendance of prescribers was required. To promote standardization in the management and clinical use of cefepime, clinical pharmacists and infectious disease experts carried out a number of these trainings for clinicians about the knowledge and clinical use of antibacterial drugs and their management through the organization of a sub-committee of the DTC, the Antibacterial Drug Management Team. For cycle A, a data collection chart was designed and approved by the Antibacterial Drug Management Team in the hospital. The chart included the items of demographics, type of surgical procedure, drug history, drug allergy, choice of antibiotic drug, dose, time of administration, number of doses, medications combined with cefepime, and replacement drugs. Conducted under the new system, a prospective (cycle B) study on 111 hospitalized patients with a cefepime treatment intervention selected at random was performed from January 2012 to May 2012. In cycle B, the planned drug therapy was evaluated before the patient received the first dose and all items were recorded as mentioned for cycle A. During the prospective study, most of the doctors were the same as those present during June to October 2011. According to the DUE criteria, the usage pattern, adverse effects, and cost-effectiveness of cefepime were compared between cycle A and cycle B.

### Inclusion and exclusion criteria

The inclusion criteria included those who received cefepime during their hospitalization in the study periods. For those with repeated cefepime courses, an interval of less than 15 days was considered as a single administration, while an interval of more than 15 days was considered as multiple administrations. The exclusion criteria were not specific.

### Definitions

**Figure 1:**
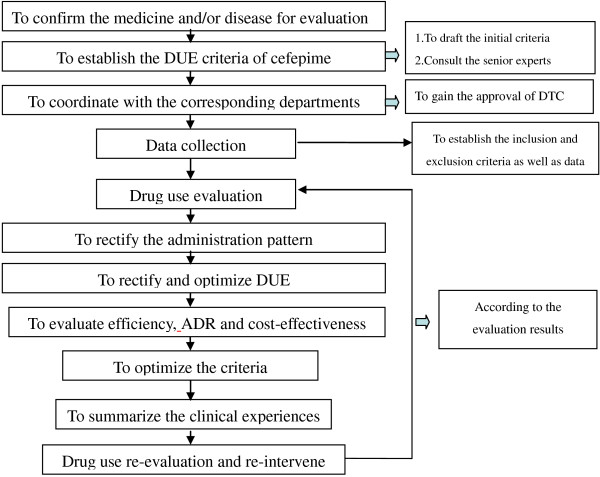
The diagram of the drug use evaluation of cefepime.

The DUE was performed according to the guidelines published by the ASHP [8]. In brief, the DUE consisted of the following eight steps: 1. to confirm the medicine and/or disease for evaluation; 2. to establish the DUE criteria and indications; 3. to gain the approval of the medical authorities (such as the hospital and/or the DTC); 4. to collect the data for drug use; 5. to rectify the drug administration pattern through education intervention; 6. to complete the second-round evaluation of the medicine; 7. to optimize the drug administration pattern and methods; and 8. to rectify the evaluation criteria and summarize the clinical experiences. The detailed steps of the DUE for our study are summarized in Figure 1.

According to the literature [9-13], the evaluation criteria for drug use include the following information: 1. diagnosis standards; 2. contraindications of the drug; 3. drug interaction standards; 4. drug administration standards; 5. treatment duration and drug dosage; 6. drug dosage per day; and 7. appropriate or inappropriate standards. Besides these standards, some other evaluation indices are needed to identify the accuracy of the collected data.

### Data analysis

The collected data were cleaned, categorized and analyzed using SPSS for Windows version 15.0. Proportions were compared using the *χ*^2^ or Fisher’s exact test, where appropriate. Continuous variables were compared by the Student’s *t*-test. All P values were two-tailed, and P < 0.05 was considered statistically significant.

### Ethics

This study was approved by the Department of Health of Anhui Province in China and the Ethics Committee of The First Affiliated Hospital of Bengbu Medical College. The need for informed consent for this study was waived by the ethics committee.

## Results

### Study population and patient characteristics

From hospitalized patients with infectious diseases, a total of 207 patients in cycle A and cycle B were identified according to the DUE criteria. In the study, the median age was 40.2 ± 15.3 years with an inter-quartile range from 5 months to 83 years in cycle A, while in cycle B the median age was 39.3 ± 14.6 years with an inter-quartile range from 8 months to 80 years. The ratios of men to women were 50:46 and 61:50, respectively. No significant differences were noted between the clinical data (P > 0.05).

After educating the professionals, Table 1 shows that the correct indication rate in cycle B was 94.59% compared with 84.38% in cycle A. Professionals demonstrated improved awareness for performing bacterial culture and sensitivity tests, as shown in Table 1, with 88.29% in cycle B compared with 65.22% in cycle A. With regards to drug monitoring and dosage, aspects that were improved in cycle B compared with cycle A included life index record (including basic patient characteristics such as height, body weight, body temperature, blood pressure, etc.), drug skin test, solvent selection (the choice of IV solution to use as the carrier of the cefepime infusion), route of administration, and compatibility. The significantly improved items in cycle B were blood examination (88.54% in cycle A and 97.30% in cycle B, P < 0.05), liver function monitoring (78.12% and 90.99%, respectively, P < 0.05), renal function monitoring (75.00% and 96.40%, respectively, P < 0.05), dose and duration (72.92% and 90.09%, respectively, P < 0.05), dosing frequency (72.92% and 90.09%, respectively, P < 0.05) and correct medication combinations (62.37% and 82.18%, respectively, P < 0.05). With regard to replacement drugs, there was no significant improvement in cycle B (P > 0.05). Table 1 shows that most items showed improved outcomes in cycle B, although a few items showed no significant improvement.

**Table 1:** Drug use evaluation of cefepime in 96 and 111 patients during cycle A and cycle B, respectively

**Items**	**Number of patients (of 96) consistent with the standards in cycle A**	**Number of patients (of 111) consistent with the standards in cycle B**
Indication	81 (84.38%)	105 (94.59%)
Drug monitoring		
Life index record	96 (100%)	111 (100%)
Blood examination	85 (88.54%)	108 (97.30%)
Liver function monitoring	75 (78.12%)	101 (90.99%)
Renal function monitoring	72 (75.00%)	107 (96.40%)
Bacterial culture and sensitivity test	60 (65.22%)*	98 (88.29%)
Drug skin test	96 (100%)	111 (100%)
Dosage		
Dose and duration	70 (72.92%)	100 (90.09%)
Dosing frequency	70 (72.92%)	100 (90.09%)
Solvent selection	96 (100.00%)	110 (99.10%)
Route of administration	96(100.00%)	111 (100.00%)
Compatibility	95 (98.96%)	111 (100.00%)
Replacement drugs	84 (87.50%)	104 (93.69%)
Combined medication (drug-drug interaction)	58 (62.37%)*	89 (80.18%)
Adverse drug reactions	8 (8.33%)	7 (6.31%)
Outcome	83 (91.21%)	102 (95.33%)

*Some cases could not be evaluated, and the percentage was calculated from the cases that could be evaluated.

Most prescribers demonstrated appropriate knowledge regarding cefepime’s prescribing, giving it for the right indications and in proper doses. The appropriate dosing frequency is every 8 h or 12 h, yet dosing frequency was performed less well in both cycles and the most common error of dosing frequency and dosage was one day’s dose IV every 24 h (Table 1). Cefepime has poor oral absorption and poor stability. Hence dosage was most commonly administered by injection. The prescribers were asked their opinions on whether the therapy was cost-effective. An average of about 80% of the prescribers in both cycles were judged to be using the therapy in a cost-effective way, which suggests that cefepime use was appropriate (Table 2). The study suggests that cefepime was being used appropriately for the right indications in the majority of patients in cycle B following the educational intervention (Table 1) and Table 2 suggests that the cost-effectiveness was the same in cycles A and B, so that the intervention had no effect on this.

**Table 2:** Cost-effectiveness of therapy as assessed by 38 prescribers during cycle A and cycle B

**Cost-effective**	**Number and percentage in cycle A**	**Number and percentage in cycle B**
Yes	30 (78.95%)	31 (81.58%)
No	5 (13.16%)	5 (13.16%)
Sometimes	3 (7.89%)	2 (5.26%)

### Medications combined with cefepime

Combinations of cefepime with fluoroquinolones and macrolides were widely used in the project. Triple therapy is generally used to cure severe infections or mixed infections, which two antimicrobials alone cannot control, such as with anaerobic bacteria or fungal infections. In cycle A, irrational combinations using cefepime were documented; for example, five patients had peri-operative period prophylactic cefepime treatment in combination with a quinolone antibiotic. After the education intervention, the results in Table 1 show that medications combined with cefepime tended to be more rational, with appropriate combinations in 89 (80.18%) out of 111 in cycle B compared with 58 (62.37%) out of 96 in cycle A.

### Adverse drug reactions

The most common adverse reactions with cefepime were nausea (2.42%), skin rash (2.42%), headache (1.44%), and diarrhea (0.48%), as shown in Table 3. Both Tables 1 and 3 show reduced adverse drug reactions in cycle B after the educational intervention.

**Table 3:** Adverse effects during cycle A and cycle B

**Adverse effects**	**Number and percentage in cycle A**	**Number and percentage in cycle B**
Headache	2 (2.08%)	1 (0.90%)
Nausea	2 (2.08%)	3 (2.70%)
Skin rash	2 (2.08%)	3 (2.70%)
Diarrhea	1 (1.04%)	0 (0%)
Other	1 (1.04%)	0 (0%)
None	88 (91.67%)	104 (93.69%)

### Resistance

Acquired microbial resistance to cefepime within healthcare facilities such as some hospitals is a growing problem [14]. The dosage, dosing frequency, duration of therapy and alternative drugs are factors contributing to the increased resistance. As shown in Table 1, compared with cycle A, rates of appropriate dosage, dosing frequency, duration of therapy and alternative drugs improved significantly in cycle B.

## Discussion

The results of the present study show that the usage pattern, adverse effects and cost-effectiveness of cefepime can be improved significantly by conducting a DUE program. An effective and rational system of cefepime use was established in the First Affiliated Hospital of Bengbu Medical College.

In the study, data were collected according to DUE criteria to assess the clinical appropriateness, cost-effectiveness and effective use of the drug therapy. The study that we conducted showed that cefepime is the drug of choice for severe gram-negative bacterial infections caused by susceptible aerobic bacteria (including *Pseudomonas aeruginosa*) and is being used in the majority of the public sector teaching hospitals according to its clinical indications, but not for infections caused by suspected anaerobic bacteria, enterococci, or pivmecillinam-resistant staphylococci, etc. Cefepime therapy proved to be cost-effective. Most hospitals exercise special control over this drug and its off-label use is prohibited in many hospitals in China. Thus cefepime used for prophylaxis was not listed in the criteria of this study or of the ASHP. In our study, justifications for prescribing cefepime were: 1. treatment of moderate and severe infections caused by suspected gram-negative bacteria or mixed aerobic bacteria (including *Pseudomonas aeruginosa*), but not for infection caused by suspected anaerobic bacteria, enterococci or pivmecillinam-resistant staphylococci; 2. alone or in combination with other antibacterial drugs to treat moderate and severe infections caused by sensitive bacteria including: lower respiratory tract infection (pneumonia and bronchitis); simple lower urinary tract infection and complicated urinary tract infections (including pyelonephritis); uncomplicated skin and skin and soft tissue infections; complicated intra-abdominal infection (including peritonitis and biliary tract infection in obstetrics and gynecology); sepsis; endocarditis for adults and 2-month to 16-year-old children; 3. empirical treatment of patients with neutropenia associated with fever; and 4. empirical treatment of the above suspected infections, although not recommended as the first-line treatment drug, unless other antimicrobials are ineffective. Compared with the ASHP criteria for cefepime, there were some adjustments in our criteria [14-16]. First, it is not recommended for osteomyelitis because of its lower concentration in the bone marrow. Second, bacterial culture and drug sensitivity tests are not widely used clinically, so these results are unable to guide clinical use. It is also very important to use cefepime empirically for the treatment of neutropenia and fever, which was added to our criteria.

The growing resistance of bacteria to antimicrobials, including cefepime, has been shown to be a very important problem. The following bacteria are associated with varying degrees of resistance to cefepime: *Staphylococcus aureus*, *Staphylococcus epidermidis*, *Streptococcus faecalis*, some even up to 100%, and *Acinetobacter baumannii* and *Escherichia coli* up to 97.3% [17,18]. With cefepime as the specific designated antibiotic drug for serious gram-negative bacteria or mixed aerobic bacterial infections, there is the growing prospect that resistance will result in a return to the days when fatal bacterial infections were common. As is well known, the dosage, dosing frequency, duration of therapy and alternative drugs are contributing factors to increased resistance. In cycle A, we found that excessive doses or inappropriate dosing frequencies of cefepime regimens were used. The most common dose and dosing frequency error was that an entire day’s dose was used in patients at one time, probably because of staff nurses’ workload. Irrational alternative drugs and unnecessary, improper and prolonged use were also present because of incorrect prescriptions from professionals. Through our cefepime use intervention to educate professionals to use cefepime correctly, improved outcomes for many of these problems were observed in cycle B.

The most common adverse reactions to cefepime are headache, nausea, skin rash and diarrhea [19,20]. The adverse effects induced by cefepime therapy are shown in Table 3. In cycle B, we found that if due care was given and/or the infusion time was 15–30 min, the adverse effects could be avoided. However, the staff nurses are in a hurry because of their workload and they usually administer the drugs rapidly, which results in adverse reactions. To strengthen safety supervision, adjustments were given by us to them, including: first, increasing the observation and treatment of rare adverse reactions (such as toxic nephrosis and renal dysfunction), while emphasizing the detection and specific treatment measures for different types of adverse reactions; second, promoting clinical experience to prevent the possibility of adverse reactions occurring (such as the use of antibiotics in patients along with the timely use of intestinal microecological preparations, according to the drug instructions) [16]. The reasons for adding these items are that: 1. the ADR reporting system should be made more efficient to minimize the incidence of adverse effects during the therapy; 2. in our healthcare setup, the majority of the ADRs are unnoticed; 3. patients should be given sufficient information regarding their medicines so that they can identify any abnormal finding in their course of therapy [21,22]; 4. the effectiveness of cefepime therapy can be enhanced and the adverse effects can be avoided to some extent by more vigilant and positive attitudes among prescribers, nurses and pharmacists. This requires a more collaborative practice among physicians and pharmacists so that pharmacists can guide physicians to the rational prescription and safer use of medicines.

## Conclusions

Comparisons were made between the two periods of cefepime use, between which we set up more reasonable cefepime use criteria in the First Affiliated Hospital of Bengbu Medical College, Anhui, China. Our study suggests that we detected potential problems and improved cefepime use through the DUE program. We believe that the new criteria will assist professionals to use cefepime more correctly in the future and that the DUE program will become a model of hospital pharmacy care and part of the plan for continuous improvements to the quality of health care in China.

## Competing interests

The authors declare that they have no competing interests.

## Authors’ contributions

QPS: study design and conception, data/statistical analyses, drafting the manuscript; MLY and FD: study design, data/statistical analyses; RS, YL and HYY: data collection and statistical analyses. All authors contributed to writing the final manuscript. All authors read and approved the final manuscript.

## Pre-publication history

The pre-publication history for this paper can be accessed here:

http://www.biomedcentral.com/1471-2334/13/160/prepub
